# HyperVision-HSI: a classification-guided spectral-spatial decoupling framework for adaptive multi-category fruit SSC detection

**DOI:** 10.3389/fpls.2026.1771621

**Published:** 2026-03-11

**Authors:** Tongtong Dong, Dongpo Wei, Longjie Li, Yujuan Fang, Yuzhen Liu, Fangyan Zhang, Guangyuan Wang

**Affiliations:** 1 College of Mechanical Engineering, Shandong Huayu University of Technology, Dezhou, China; 2 College of Mechanical and Electrical Engineering, Xinjiang Agricultural University, Urumqi, China

**Keywords:** cross-species sugar content prediction, dynamic ROI localization, hyperspectral imaging, nondestructive detection, spectral-spatial decoupling

## Abstract

In the non-destructive detection of soluble solids content (SSC) across multi-category fruits, hyperspectral imaging (HSI) often faces challenges such as dynamic localization distortion of biological feature regions and significant inter-species optical heterogeneity. This study proposes HyperVision-HSI, a classification-guided spectral-spatial adaptive decoupling framework. By integrating dynamic ROI localization, dual-channel spectral calibration, and a category-aware model invocation architecture, the framework achieves precise feature extraction and matching for multi-category samples. Utilizing real-time classification information as a decision-making shunt, the framework automatically triggers species-adaptive thresholds to extract high-purity ROIs for grapes, tomatoes, and Xiangli pears—three species with markedly different optical properties. It then dynamically invokes pre-optimized specialized regression models (Grapes: KRR; Tomatoes: BRR; Xiangli Pears: Lasso), effectively addressing the feature dilution problem encountered by single models when processing heterogeneous samples. Experimental results demonstrate that the system achieves SSC prediction errors (RMSE) as low as 0.62°Brix, 0.32°Brix, and 0.37°Brix on independent test sets for the three fruit types, respectively, with a single-frame processing time of less than 1 second. The modular architecture and high scalability of HyperVision-HSI provide a rigorous adaptive technical pathway for the automated detection of multi-category agricultural phenomics in diverse scenarios.

## 1 Introduction

In the process of agricultural industrialization, real-time non-destructive detection of internal fruit quality is crucial for improving post-harvest sorting efficiency and ensuring the value of agricultural products (Anjali et al., 2024; Liu et al., 2025; He et al., 2022; Akter et al., 2024; Xu et al., 2024). As the core indicator for evaluating sugar content, the accurate and rapid detection of soluble solids content (SSC) directly influences harvesting decisions, market grading, and consumer satisfaction (Grabska et al., 2023; Yu et al., 2025; Hu et al., 2024). Traditional chemical detection methods (e.g., refractometers, high-performance liquid chromatography) (Zhang et al., 2022; Noguera et al., 2022; Jaywant et al., 2022; Borba et al., 2021) rely on destructive sampling, which inherently suffers from low efficiency, high cost, and the inability to achieve full-sample screening. Subsequent single-point spectroscopy technology, while realizing non-destructive detection (Fang et al., 2020; Li et al., 2020; Égei et al., 2022), still requires fruit-by-fruit sampling and analysis, resulting in severely limited efficiency that is difficult to meet industrial demands. In recent years, hyperspectral imaging technology, leveraging the ad-vantage of integrating spectra and images (Gao and Xie, 2024; Luo et al., 2024; Tian et al., 2023), has achieved breakthroughs in integration with computer vision and electronic technology. Low-cost IoT hyperspectral prototypes constructed using 3D-printed components and commercial electronic devices (e.g., Raspberry Pi, Raspicam NoIR) (Vignati et al., 2025) have enabled distributed monitoring in the 400–1000 nm band. Additionally, combining deep learning algorithms (e.g., convolutional neural networks) and multi-sensor fusion architectures has improved detection accuracy (Wan et al., 2025), while optimizing data acquisition and processing workflows through computational im-aging technology integrated with CMOS chips (Sun et al., 2024), providing a new pathway for non-destructive detection. However, it still faces two core bottlenecks in cross-species sugar content detection: (1) dynamic localization distortion of biological feature regions in complex scenarios, leading to spectral data being susceptible to interference from fruit stems, fruit navels, uneven peel color, specular reflection, and dark noise; (2) spectral-spatial coupling effects caused by sensor dark current drift and light fluctuations, which significantly degrade data quality and restrict model generalization.

Current research mainly focuses on optimizing spectral equipment and improving machine learning algorithms, but has not fundamentally addressed the aforementioned issues. In terms of target localization, traditional studies mostly rely on manual ROI de-lineation. For example, Kanwal et al (Kanwal et al., 2025). used Spectronon software to manually extract ROIs from the side or pulp regions of lychees and mangoes. Their subjectivity leads to empirical biases among different researchers in defining “complete sides” or “avoiding peel regions,” resulting in blurred ROI boundaries and spectral data prone to noise contamination. Benelli et al. (Benelli et al., 2022) extracted kiwifruit ROIs via k-means clustering, which can only distinguish between the background and fruits but exhibits poor robustness to com-plex surface features such as shadows and skin spots, often leading to the erroneous exclusion of edge regions. Studies by Lyu et al (Lyu et al., 2024). and Qiu et al (Qiu et al., 2024). attempted optimization by excluding shadows but still relied on static definitions of “middle regions” or “whole fruit regions,” failing to consider significant inter-species heterogeneity in epidermal structure and optical properties—such as the thin waxy layer of grapes (weak specular reflection), smooth cuticle of tomatoes (local high-reflection patches), and rough epidermis of Xiangli pears (diffuse reflection)—resulting in insufficient dynamic adaptability in cross-species scenarios. Li et al (Li et al., 2023). directly used the “entire fruit region” as the ROI to extract average spectra, but spectral data were easily mixed with shadow and epidermal noise due to the failure to distinguish complex features such as uneven oil cell distribution in Tribute citrus and grape skin blemishes. Critically, most existing studies focus on single fruit varieties such as apples (Pang et al., 2021) or citrus (Kim et al., 2024), lacking exploration of joint modeling for species with distinct optical properties (e.g., grapes, tomatoes, Xiangli pears). This limitation directly restricts technical universality: grapes have a thin waxy layer and weak specular reflection; tomatoes have a smooth cuticle leading to local high-reflection patches; Xiangli pears have a rough epidermis with dense stomata, resulting in globally diffuse re-flection. Inter-species differences in peel structure and color render traditional ROI localization methods inadequately adaptable in cross-species scenarios, causing spectral data to be contaminated with substantial invalid noise. Furthermore, inherent dark current drift and light fluctuations in hyperspectral imaging systems exacerbate spectral-spatial coupling effects, manifested as baseline drift and reduced signal-to-noise ratio, which severely weaken model robustness and cross-species generalization.

To address these bottlenecks, this study proposes the HyperVision-HSI framework, a cross-species SSC non-destructive detection paradigm based on adaptive spectral-spatial decoupling. By integrating dynamic ROI localization, dual-channel spectral radiometric calibration, and a classification-guided lightweight model invocation architecture, the framework constructs a full-process technical chain of “classification-localization decoupling-feature analysis-prediction analysis.” In the dynamic ROI localization stage, the YOLOv8n-seg instance segmentation model is used for real-time target detection and mask generation. Combined with centripetal scaling of mask centroids (scaling factor s = 0.18) and species-adaptive grayscale thresholds (grapes: 30–150; tomatoes: 60–180; Xiangli pears: 60–210), it effectively suppresses peel specular reflection and dark noise, extracting high-purity biological feature regions. In the spectral-spatial decoupling stage, sensor noise is eliminated through radiometric calibration and dual-channel calibration, and input data are optimized via maximum normalization, significantly enhancing the anti-interference capability of spectral data. Experimental verification shows that HyperVision-HSI achieves SSC prediction errors (RMSE) as low as 0.62°Brix, 0.32°Brix, and 0.37°Brix on independent test sets of grapes, tomatoes, and Xiangli pears, respectively, with a single-frame processing time of less than 1 second. It effectively meets the dual requirements of real-time performance and accuracy in agricultural industrial scenarios, providing an efficient and feasible solution for cross-species non-destructive detection.

## 2 Materials and methods

### 2.1 Overview of the experimental method

**Figure 1:**
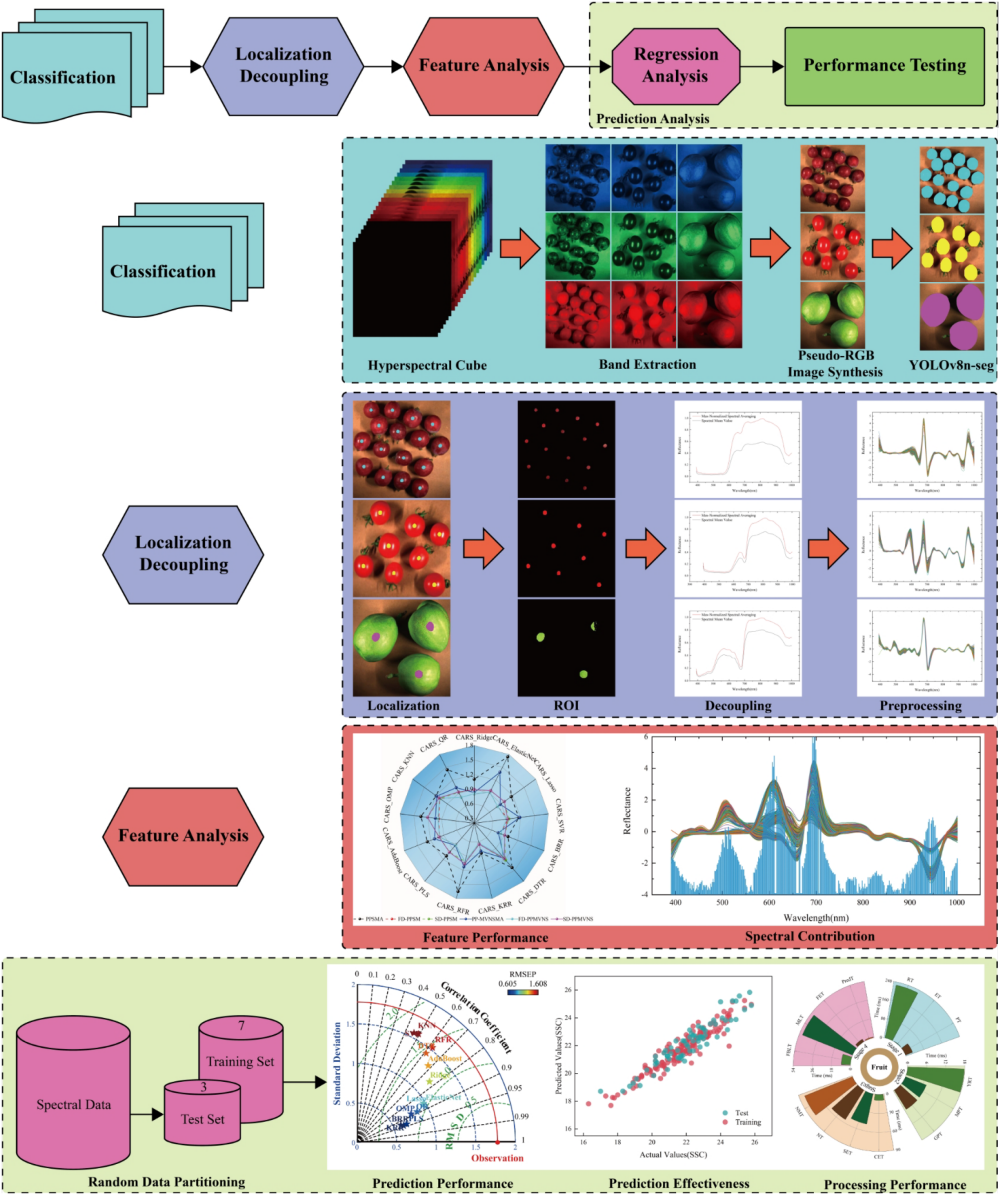
Flowchart of experimental methods.

This study proposes the HyperVision-HSI framework, which takes “classification-localization decoupling-feature analysis-prediction analysis” as its core technical chain. Its complete workflow is illustrated in **Figure 1**, encompassing four key stages. First, hyperspectral cube data of grapes, tomatoes, and Xiangli pears are collected, and data from adjacent bands (R: 650 nm, G: 550 nm, B: 450 nm) are extracted to synthesize pseudo-RGB images. Subsequently, the YOLOv8n-seg instance segmentation model is utilized to achieve target classification and mask generation. High-purity ROI regions are extracted by suppressing specular reflection and dark noise through centripetal scaling of the mask centroid (scaling factor s = 0.18) and species-adaptive grayscale thresholds (grapes: 30–150; tomatoes: 60–180; Xiangli pears: 60–210). Furthermore, anti-interference dual-channel input data are constructed based on raw spectra and maximum-normalized spectra. Finally, optimal regression models are dynamically invoked according to fruit categories (grapes: Kernel Ridge Regression/KRR; tomatoes: Bayesian Ridge Regression/BRR; Xiangli pears: Lasso) to realize accurate SSC prediction. Through an adaptive decoupling mechanism, the framework effectively overcomes dynamic localization distortion of biological features and spectral-spatial coupling interference, providing a unified interface for cross-species SSC detection.

### 2.2 Experimental materials and data collection

**Figure 2:**
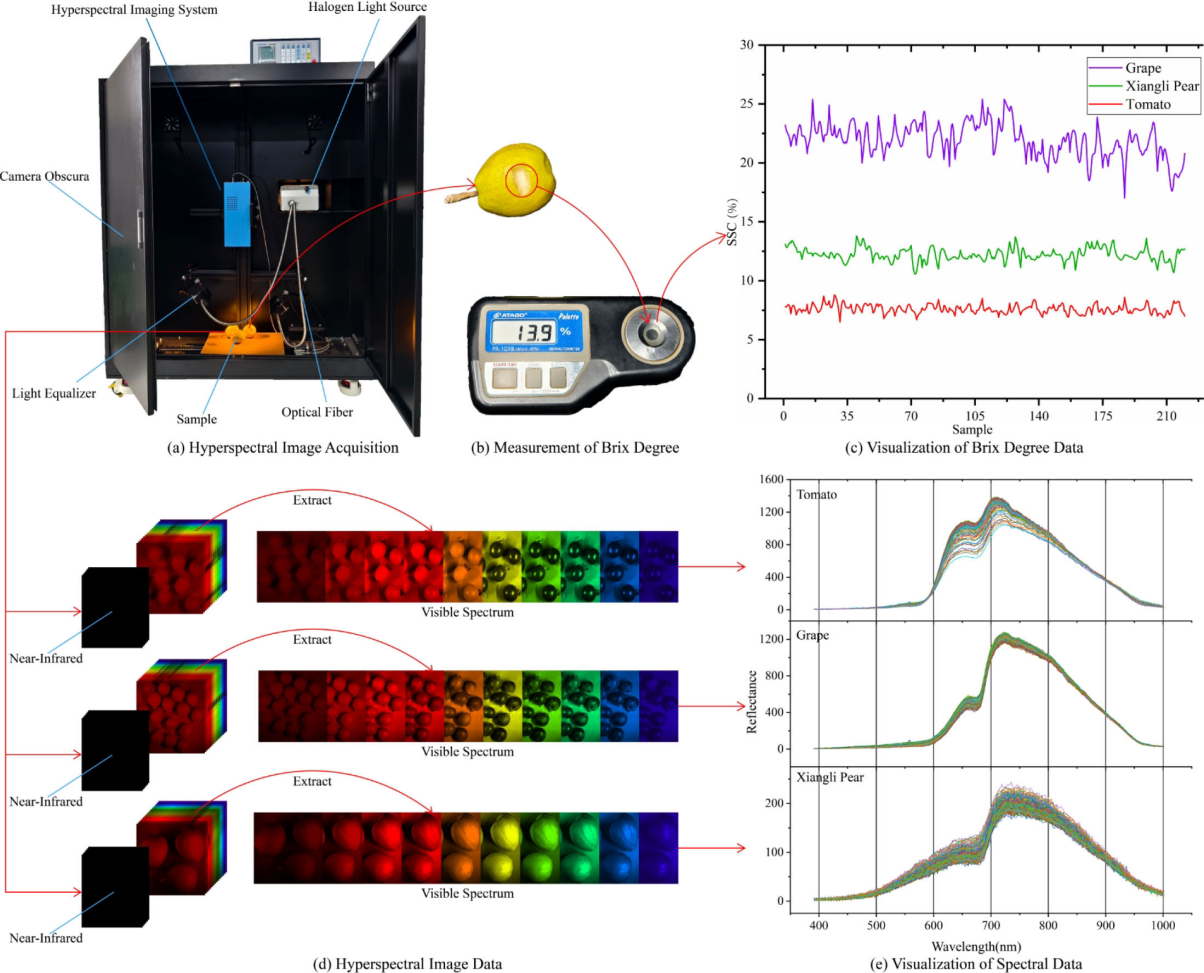
Flowchart of data collection. **(A)** Hyperspectral data collection system, **(B)** SSC measuring instrument, **(C)** Ground-truth SSC, **(D)** Hyperspectral cube data, **(E)** Fruit spectral data.

Three typical horticultural fruits—grapes, tomatoes, and Xiangli pears—were select-ed as experimental subjects. Their distinct differences in epidermal optical properties and fruit colors significantly influence specular reflection suppression, dark noise control, and ROI localization accuracy in hyperspectral imaging, thereby exhibiting representative experimental value. Grapes possess a thin waxy layer with low specular reflection intensity; tomatoes feature a smooth cuticle characterized by high local reflection intensity and small reflection patch areas; Xiangli pears have a rough epidermis with dense stomata, showing globally diffuse reflection. Differences in fruit color and peel properties result in significant brightness differentiation in visible light imaging regions (**Figure 2D**). A total of 670 samples with uniform maturity were collected for the experiment (226 grapes, 221 tomatoes, and 223 Xiangli pears), with the equatorial diameter ranges of 18–22 mm for grapes, 20–26 mm for tomatoes, and 65–80 mm for Xiangli pears.

The fruits were horizontally placed on a standard yellow background cardboard, and a stable illumination environment was provided by a 150 W halogen lamp equipped with dual light equalizers. Hyperspectral data acquisition (**Figure 2E**) was performed using a hyperspectral imaging system (spectral range: 390–1000 nm, totaling 346 bands, **Figure 2A**), and the raw hyper-spectral cubes were stored in ENVI format after radiometric correction. The true SSC values were measured at the equatorial region of the fruits using a portable refractometer (ATAGO PR-101α, accuracy: ± 0.1°Brix; **Figure 2B**). Each sample was measured three times independently, and the average value was adopted as the label data (**Figure 2C**).

### 2.3 Hyperspectral data preprocessing

**Figure 3:**
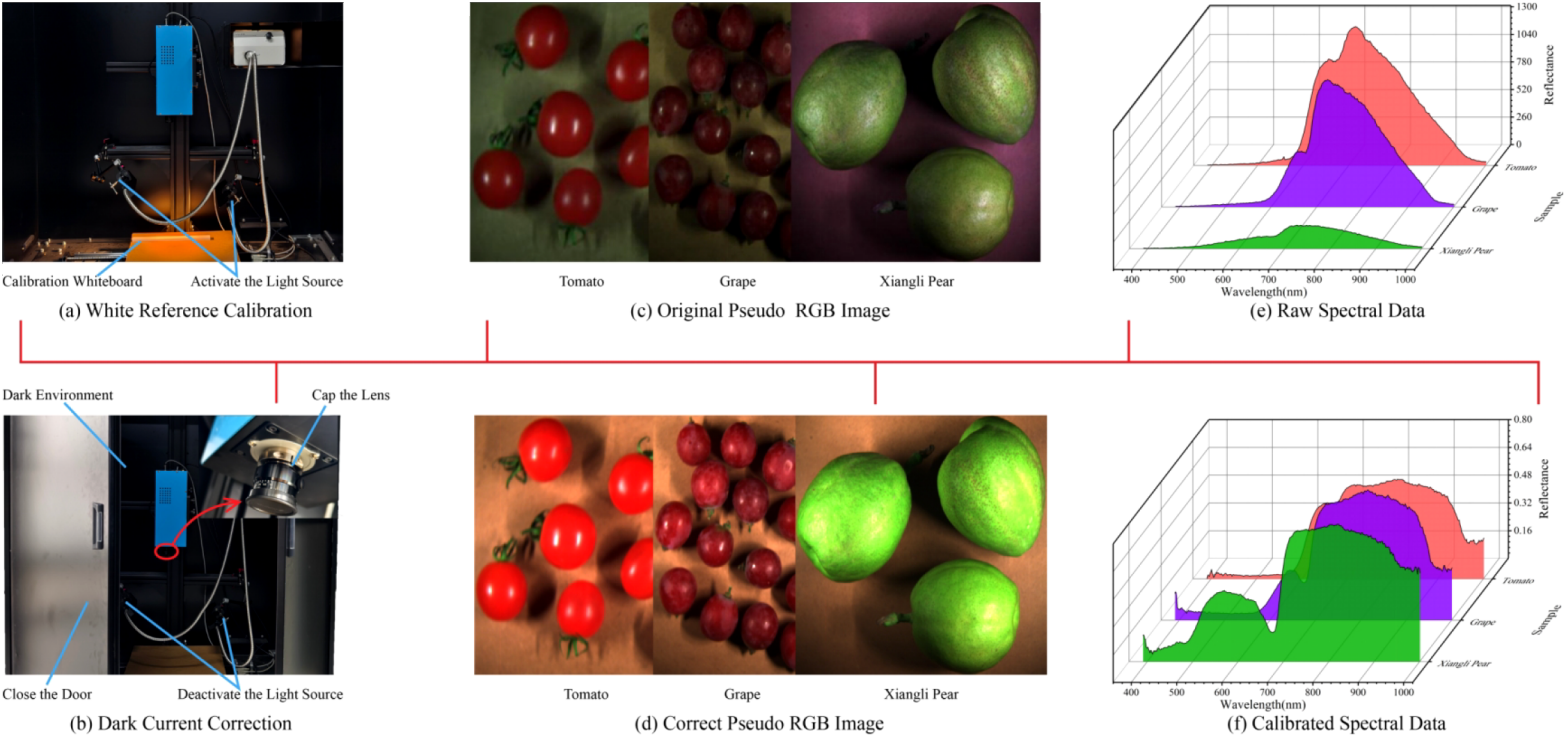
Hyperspectral data preprocessing. **(A)** Whiteboard calibration, **(B)** Dark current calibration, **(C)** Original pseudo-RGB image, **(D)** Calibrated pseudo-RGB image, **(E)** Original fruit spectral data, **(F)** Calibrated fruit spectral data.

Hyperspectral imaging systems introduce systematic noise during data acquisition due to factors such as sensor dark current drift, light source intensity fluctuations, and nonlinear responses, resulting in baseline drift and reduced signal-to-noise ratio (SNR) in spectral data, which directly impairs the accuracy and robustness of sugar content modeling. To mitigate such interference, sensor noise and ambient light contamination are eliminated through radiometric calibration and reflectance conversion, enabling the correction and conversion of the reflectance of raw hyperspectral data (denoted as $DN_{raw}(x,y,\lambda )$) (Equation 1). As illustrated in **Figures 3A**, a calibration whiteboard was placed at the center of the spectral acquisition field to obtain the whiteboard reference value $DN_{white}(\lambda )$. For **Figures 3B**, the dark box door was fully closed, the light source was turned off, and the lens cap was secured, placing the acquisition system in a completely dark environment to measure the dark current reference value $DN_{dark}(\lambda )$. The corrected spectral reflectance R(x,y,λ) was then derived through calculation. **Figures 3C, D** present the visualizations of pseudo-RGB images before and after correction, while **Figures 3E, F** show the visualizations of ROI spectral reflectance before and after correction.

(1) $$R(x,y,\lambda )=\frac{DN_{raw}(x,y,\lambda )\;-\;DN_{dark}(\lambda )}{DN_{white}(\lambda )\;-\;DN_{dark}(\lambda )}$$

R(x,y,λ): Corrected spectral reflectance at the pixel point (x,y).

$DN_{raw}\left(x,y,\lambda \right)$: Original spectral reflectance at the pixel point (x,y).

$DN_{white}\left(\lambda \right)$: Whiteboard reference value.

$DN_{dark}\left(\lambda \right)$: Dark current reference value.

### 2.4 Dynamic ROI localization and noise suppression

**Figure 4:**
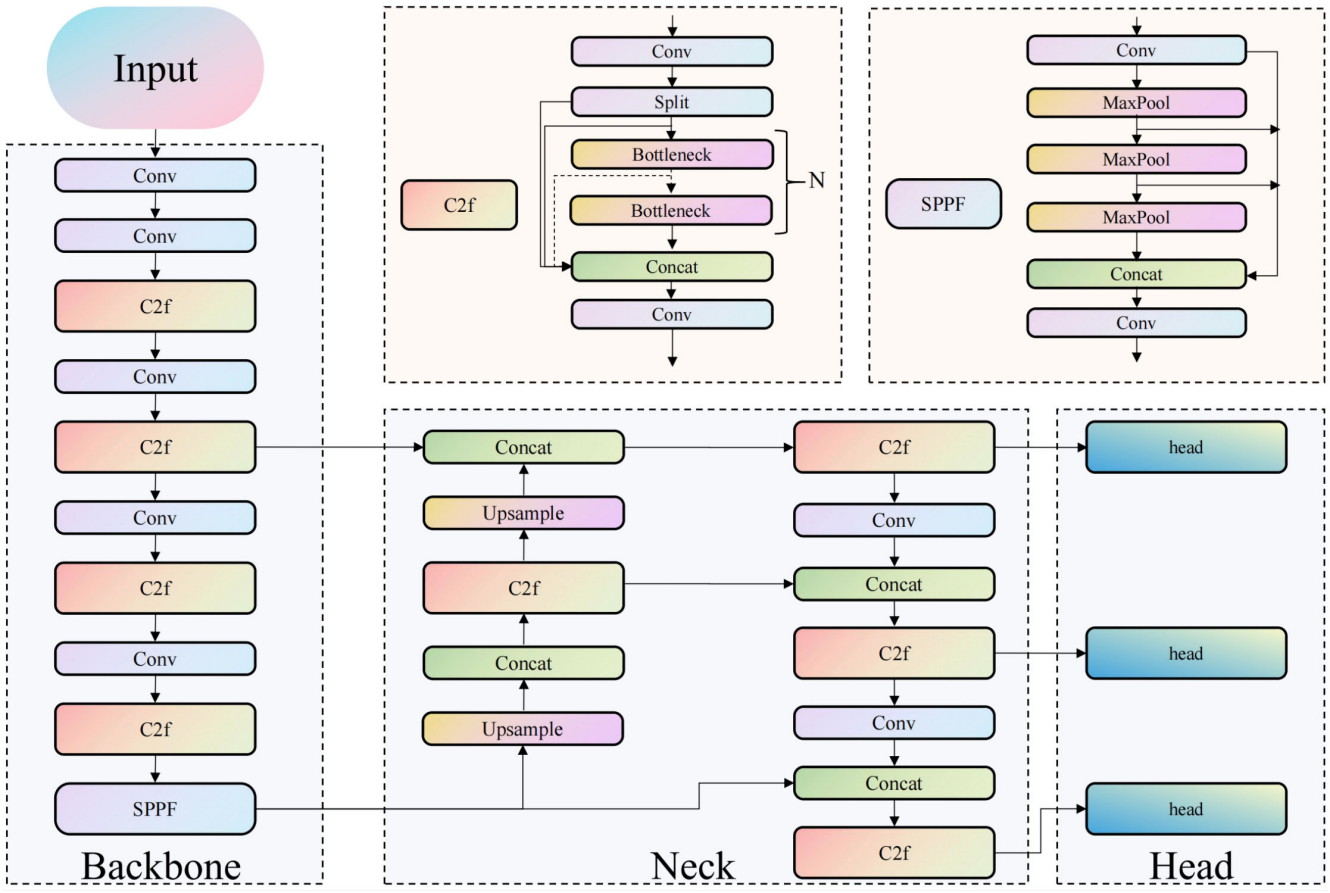
Training structure of the YOLOv8 instance segmentation model.

**Figure 5:**
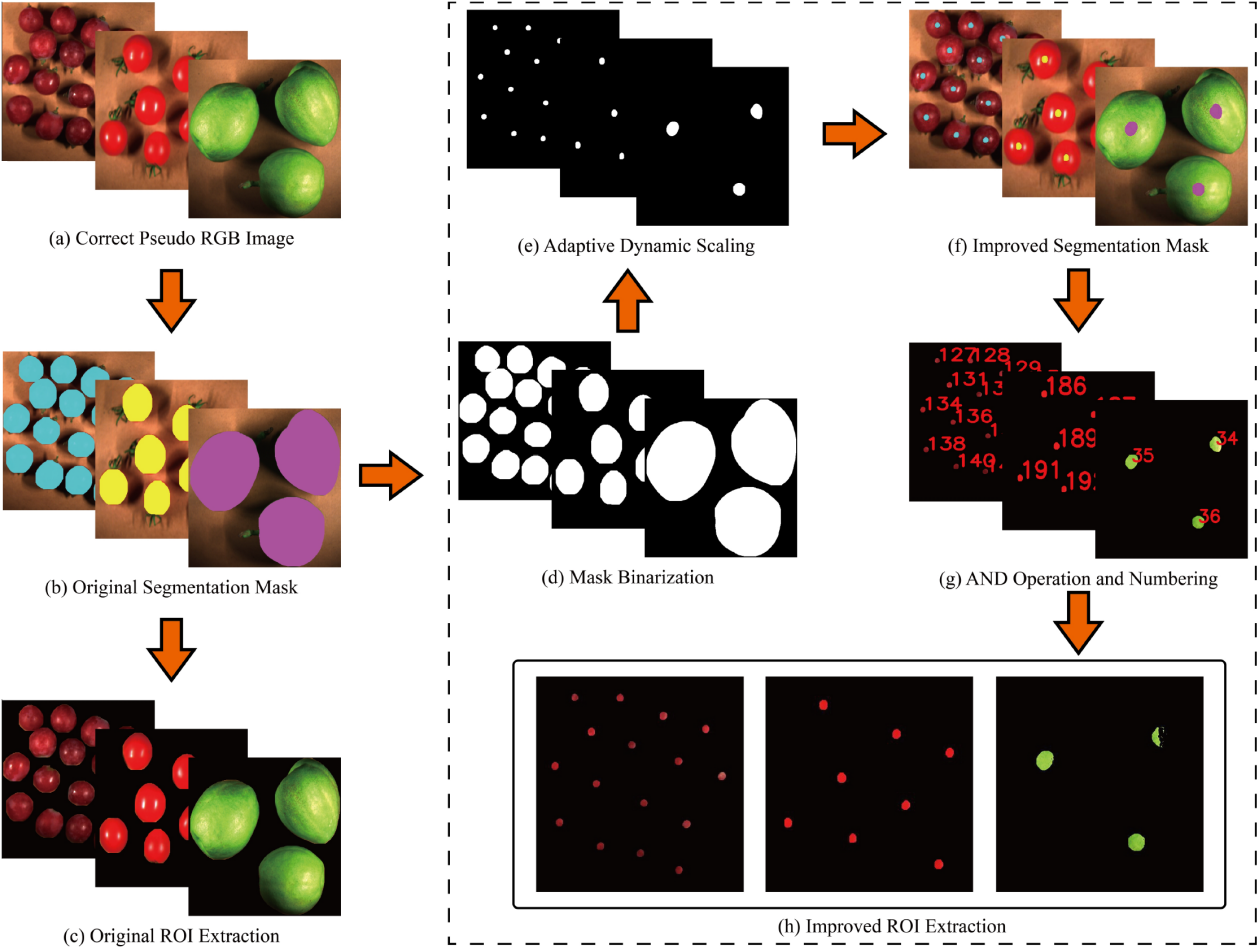
Flowchart of dynamic ROI localization **(A)** Calibrated pseudo-RGB image, **(B)** Original segmentation mask, **(C)** Original ROI extraction, **(D)** Mask binarization, **(E)** Mask scaling, **(F)** Improved segmentation mask, **(G)** Improved ROI extraction, **(H)** High-purity ROI extraction.

Real-time detection of fruit targets and dynamic ROI localization are achieved based on the YOLOv8n-seg instance segmentation model (**Figure 4**). The model undergoes non-transfer training, eliminates redundant detection boxes via non-maximum suppression, and outputs target categories, bounding boxes, and fruit contour masks. To address the issues of peel specular reflection and dark noise, geometric centripetal scaling and adaptive grayscale thresholds are adopted for interference suppression: first, the mask is binarized, and contour points are scaled centripetally in equal proportion with the centroid (xc, yc) as the reference and a fixed scaling factor s = 0.18 (Equation 2) to generate a scaled mask. The binarized scaled mask is subjected to a bitwise AND operation with the original pseudo-RGB image to extract the scaled ROI region (Equation 3), and each ROI is assigned a unique target ID to ensure accurate correspondence with the measured SSC values. Subsequently, the scaled ROI region is converted into a grayscale image (Equation 4), and species-specific OTSU thresholds are set (grapes: 30 ≤ grayscale value ≤ 150; tomatoes: 60 ≤ grayscale value ≤ 180; Xiangli pears: 60 ≤ grayscale value ≤ 210) to remove specular re-flection and dark noise pixels (Equation 5). Ultimately, the extraction of high-purity biological feature regions is realized (**Figure 5**).

(2) $$x^{\prime }=x_{c}+0.18(x-x_{c}),\;y^{\prime }=y_{c}+0.18(y-y_{c})$$

$\left(x_{c},y_{c}\right)$: Coordinates of the mask centroid.

(x,y)): Coordinates of the original mask points.

(x',y'): Coordinates after scaling.

(3) $$I_{ROI}(\;x,y\;)\;=\;\{\begin{array}{c} I_{RGB}(\;x,y\;),\;\;\;\;\;\;\;\;\;Mask\;Binary_{\left(\;x,y\;\right)}=1\\ 0,\;\;\;\;\;\;\;\;\;\;\;\;\;\;\;\;\;\;\;\;\;\;\;\;\;\;Mask\;Binary_{\left(\;x,y\;\right)}=0 \end{array}$$

$I_{ROI}\left(x,y\right)$: Scaled pseudo-RGB mask.

$I_{RGB}\left(x,y\right)$: Binarized scaled mask (1 for valid regions).

(4) $$I_{gray}\;(\;x,y\;)=\;0.229R(\;x,y\;)\;+\;0.587G(\;x,y\;)\;+\;0.114B(\;x,y\;)$$

$I_{gray}\;(\;x,y\;)$: Grayscale value of the pseudo-RGB mask pixel.

R( x,y ), G( x,y ), B( x,y ): Intensity values of the R, G, B channels of the pseudo-RGB mask pixel.

(5) $$ROI_{valid}\;=\;\left\{(\;x,y\;)\;|\;I_{gray}\;(\;x,y\;)\;\in \;\left[\alpha ,\beta \right]\right\}$$

$ROI_{valid}$: Final effective ROI region.

α: Lower grayscale threshold.

β: Upper grayscale threshold.

### 2.5 Spectral feature extraction and enhancement

**Figure 6:**
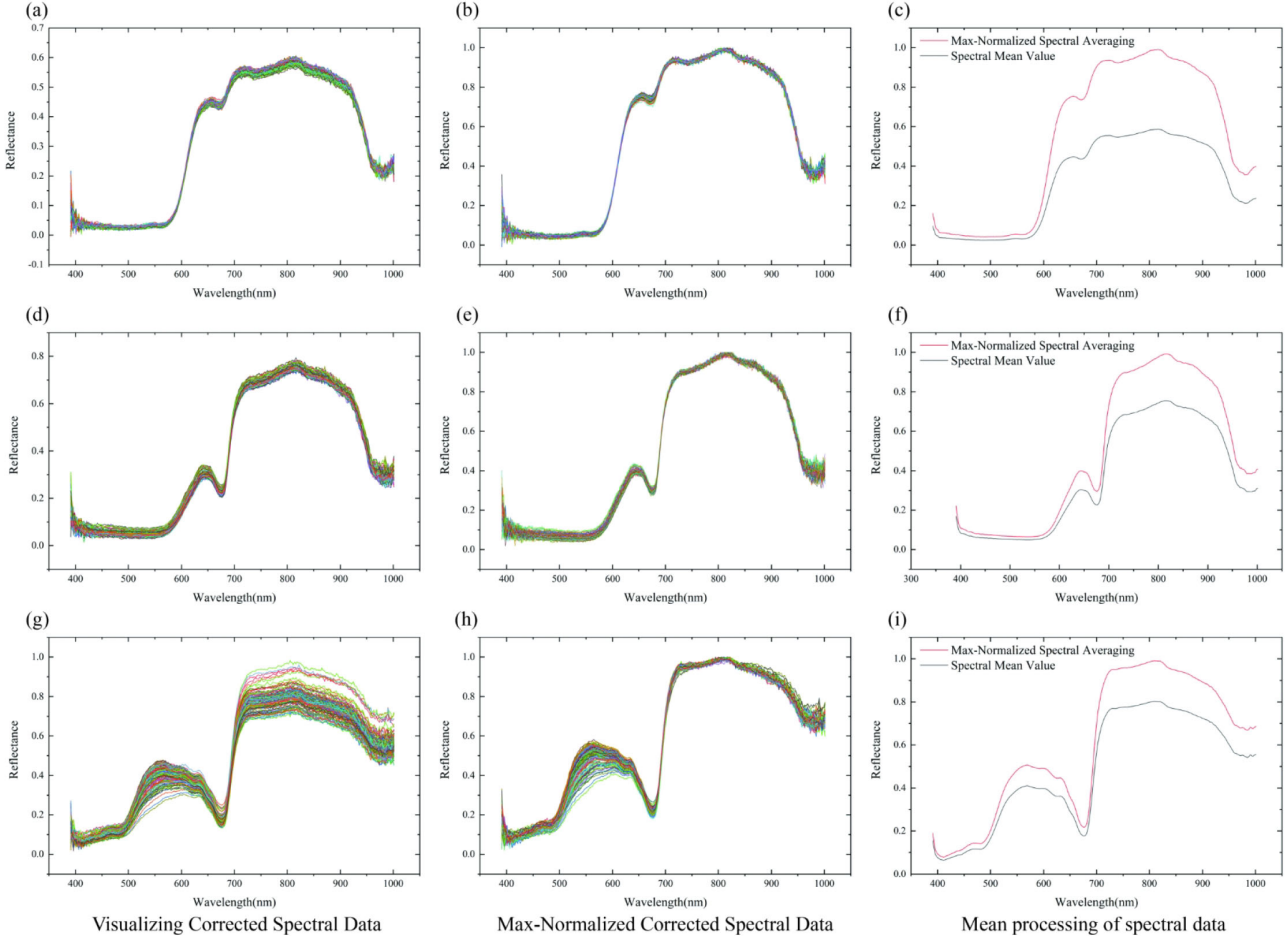
Input data optimization for sugar content prediction models. **(A)** Grape per-pixel spectral data, **(B)** Grape per-pixel normalized spectral data, **(C)** Grape original and normalized spectral means, **(D)** Tomato per-pixel spectral data, **(E)** Tomato per-pixel normalized spectral data, **(F)** Tomato original and normalized spectral means, **(G)** Xiangli pear per-pixel spectral data, **(H)** Xiangli pear per-pixel normalized spectral data, **(I)** Xiangli pear original and normalized spectral means.

Based on the valid ROIs of high-purity biological feature regions obtained through dynamic localization, pixel-wise spectral data are extracted (**Figures 6A, D, G**). Maximum normalization (Equation 6) is employed to eliminate the influence of light fluctuations, generating normalized spectral data (**Figures 6B, E, H**). Subsequently, the average spectra of these two types of spectral data are calculated separately (Equation 7), and finally, two characteristic spectral datasets—i.e., the mean of raw spectra and the mean of normalized spectra—for each ROI region are output (**Figures 6C, F, I**), providing standardized input for subsequent modeling.

(6) $$R_{norm}(x,y,\lambda )\;=\;\frac{R(x,y,\lambda )}{\max(R(x,y,\lambda ))}\;(x,y)\in ROI_{valid}$$

R(x,y,λ): Defined by Equation 1, representing the corrected spectral reflectance.

$R_{norm}\left(x,y,\lambda \right)$: Maximum-normalized corrected spectral reflectance.

(7) $$\bar{R}(\lambda )=\frac{1}{N}\sum R_{norm}(\;x,y,\lambda )\;(\;x,y)\;\in \;ROI_{valid}$$

*N*: Number of effective pixels.

$\bar{R}\left(\lambda \right)$: Average of the maximum-normalized corrected spectral reflectance in the $ROI_{valid}$ region.

**Figure 7:**
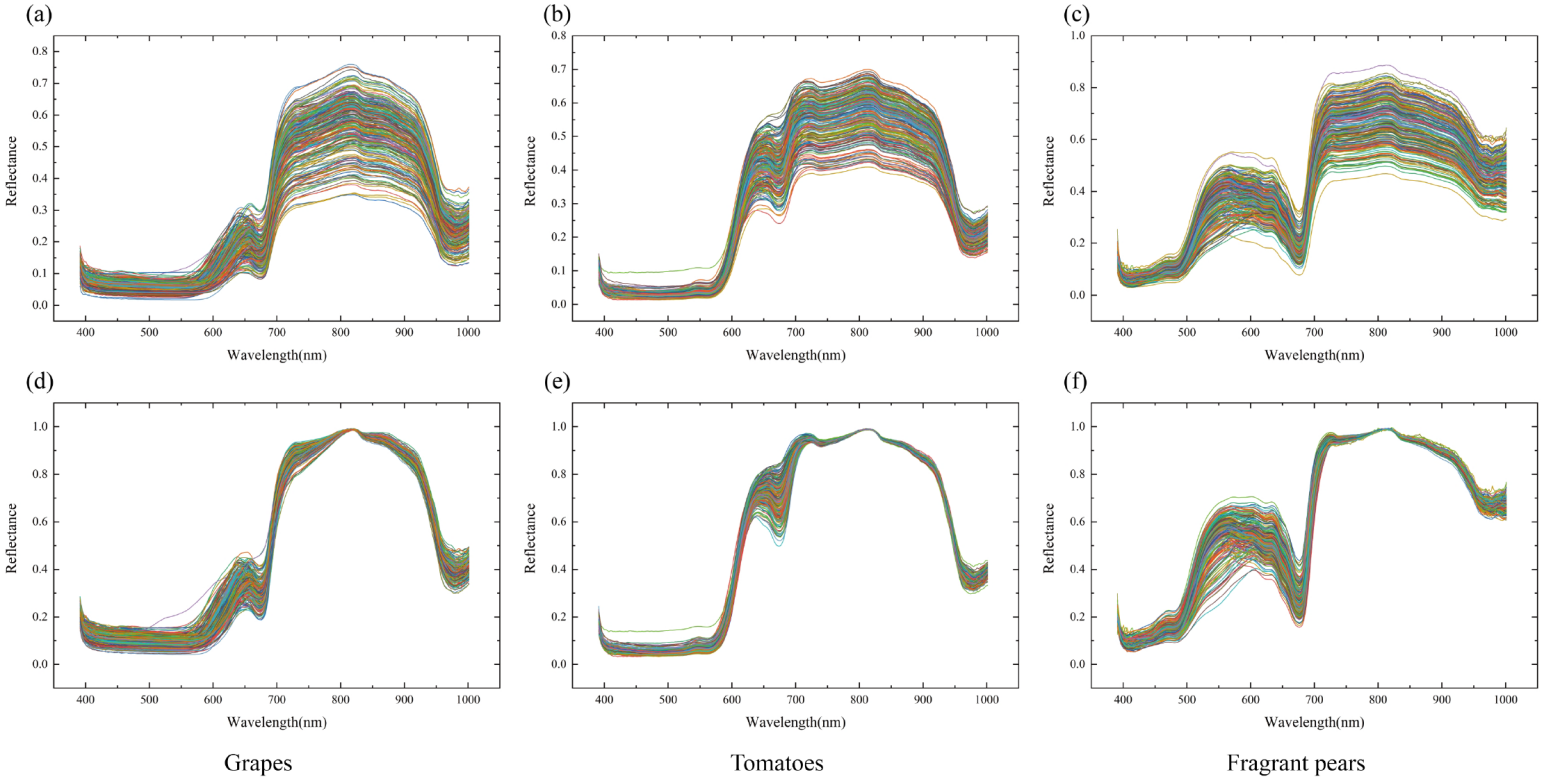
Processing of spectral data in high-purity valid ROIs. **(A)** Original grape spectral mean aggregation, **(B)** Original tomato spectral mean aggregation, **(C)** Original Xiangli pear spectral mean aggregation, **(D)** Maximum-normalized grape spectral mean aggregation, **(E)** Maximum-normalized tomato spectral mean aggregation, **(F)** Maximum-normalized Xiangli pear spectral mean aggregation.

For the valid ROIs in the high-purity biological feature regions of each sample, pixel-wise spectral data and maximum-normalized spectral data are extracted respectively for integrated analysis: **Figures 7A-C** sequentially present the results of Per-pixel Spectral Mean Aggregation within ROI (PPSMA) for grapes, tomatoes, and Xiangli pears, while **Figures 7D-F** correspondingly display the Per-pixel Maximum-Value Normalized Spectral Mean Aggregation within ROI (PP-MVNSMA) data for the three fruit species. To further enhance the discriminability of spectral features, differential preprocessing is performed on the aforementioned data: the First Derivative of Per-pixel Spectral Mean within ROI (FD-PPSM) and First Derivative of Per-pixel Maximum-Value Normalized Spectral Mean within ROI (FD-PPMVNS) features are extracted for grapes, tomatoes, and Xiangli pears, respectively; meanwhile, the Second Derivative of Per-pixel Spectral Mean within ROI (SD-PPSM) and Second Derivative of Per-pixel Maximum-Value Normalized Spectral Mean within ROI (SD-PPMVNS) feature distributions of the three fruit species are ac-quired.

### 2.6 Spectral feature selection and modeling

The Competitive Adaptive Reweighted Sampling (CARS) algorithm was employed to screen spectral feature wavelengths most relevant to SSC. All wavelength weights were initialized to 1, and feature subsets were dynamically iterated following an exponentially decreasing rule. Weights were updated by integrating 13 regression models, including Ridge Regression, Lasso Regression, Elastic Net, Support Vector Regression (SVR), Decision Tree, Random Forest, K-Nearest Neighbors (KNN), Adaptive Boosting, Bayesian Ridge Regression (BRR), Kernel Ridge Regression (KRR), Partial Least Squares (PLS), Orthogonal Matching Pursuit (OMP), and Quantile Regression. Specifically, linear models (Ridge Regression, Lasso Regression, Elastic Net, PLS, OMP, Quantile Regression) updated weights based on the absolute values of regression coefficients; support vector ma-chines (SVR, BRR, KRR) and tree-based models (Decision Tree, Random Forest, Adaptive Boosting) utilized feature importance metrics for weight updates; and KNN adjusted weights according to feature contribution degrees. Finally, the weights from all models were integrated via weighted averaging to output the optimal feature subset.

The extracted feature data were split into a 70% calibration set (i.e., training set) and a 30% prediction set (i.e., test set). Hyperparameters were optimized using five-fold cross-validation combined with grid search on the calibration set, and model stability was evaluated through 100 iterations with different random seeds. The coefficient of determination (R²) of the prediction set was adopted as the core evaluation metric, supplemented by root mean square error (RMSE) and mean absolute error (MAE) for comprehensive performance assessment. Model selection adhered to the overfitting avoidance principle: priority was given to models where the calibration set coefficient of determination (R_c_²) was higher than the prediction set coefficient of determination (R_p_²); if no such model existed, the one with the highest R_p_² was selected. Ultimately, the optimal model parameters were saved as a.pkl file for deployment.

### 2.7 System deployment and real-time performance verification

To evaluate the performance of the HyperVision-HSI framework in practical application scenarios, end-to-end system deployment and real-time performance testing were conducted in this study. Tests were carried out in a simulated production line environment, with the hardware platform configured as follows: Intel Core i9-13900K CPU @ 3.0 GHz, NVIDIA GeForce RTX 4090 GPU (24 GB VRAM), and 64 GB DDR5 RAM. The soft-ware environment was built on the Ubuntu 20.04 LTS operating system, integrated with Python 3.9. The time consumption of each link in the system processing workflow was measured using a program timer, covering the entire chain from hyperspectral cube input to the output of the final sugar content prediction value. Specifically, the measured metrics included: hyperspectral data reshaping time (RT), YOLOv8 target recognition and segmentation time (YRT), mask post-processing time (MPT), grayscale conversion time (GPT), coordinate extraction time (CET), spectral data extraction time (SET), normalization processing time (NT), raw spectral mean calculation time (RMT), normalized spectral mean calculation time (NMT), normalized spectral second derivative calculation time (NSDT), raw spectral first derivative calculation time (RFDT), feature band loading time (FBLT), model loading time (MLT), feature extraction time (FET), and prediction time (PredT).

## 3 Results

### 3.1 Performance analysis of fruit target localization and segmentation

The YOLOv8n-seg instance segmentation model achieves target detection and in-stance segmentation for three fruit types (grapes, tomatoes, and Xiangli pears) under non-transfer training conditions (input size: 640×640, confidence threshold: 0.6, IoU threshold: 0.5). As presented in **Table 1**, after 200 epochs of training on the custom dataset, the model attains a bounding box detection accuracy (Box mAP50-95) of 97.5% and an in-stance segmentation mask accuracy (Mask mAP50-95) of 94.6%. The average recall and precision on the validation set remain stably above 99%, verifying its robustness to multi-scale targets and complex fruit morphologies.

**Table 1:** Target recognition and segmentation performance.

Class	Object detection task (%)				Instance segmentation task (%)			
	Precision	Recall	mAP50	mAP50-95	Precision	Recall	mAP50	mAP50-95
All	99.2	99.1	98.6	97.5	99.2	99.1	98.6	94.6
Grapes	97.7	97.4	96.99	94.5	97.7	97.4	96.9	90.1
Xiangli pears	99.9	1	99.5	99.4	99.9	1	99.5	99.4
Tomatoes	1	1	99.5	98.5	1	1	99.5	94.2

**Figure 8:**
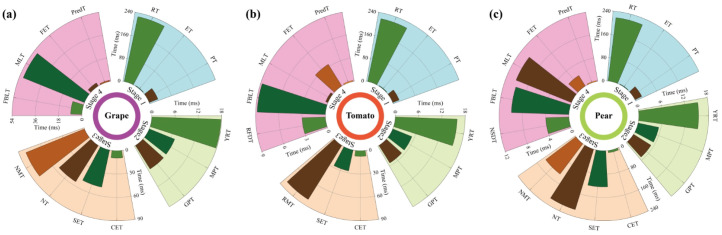
Real-time performance and time consumption analysis of HyperVision-HSI system deployment for **(A)** grapes, **(B)** tomatoes, and **(C)** Xiangli pears.

**Figure 9:**
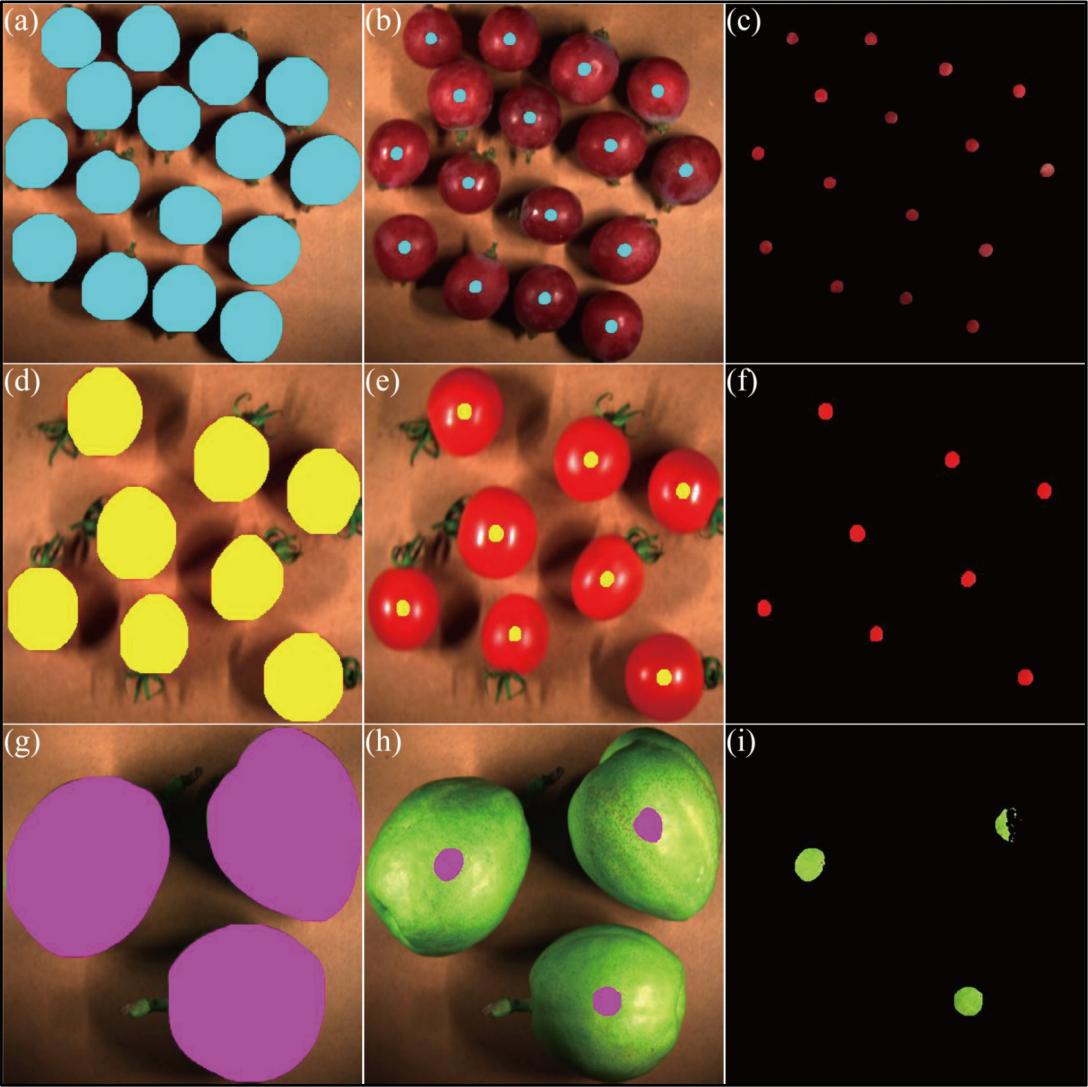
Fruit target localization and segmentation **(A)** Original mask of grapes, **(B)** Improved mask of grapes, **(C)** High-purity ROI of grapes, **(D)** Original mask of tomatoes, **(E)** Improved mask of tomatoes, **(F)** High-purity ROI of tomatoes, **(G)** Original mask of Xiangli pears, **(H)** Improved mask of Xiangli pears, **(I)** High-purity ROI of Xiangli pears.

Following the output of original masks by the model, post-processing is performed using geometric centripetal scaling (scaling factor s=0.18) and species-adaptive grayscale thresholds (grapes: 30≤grayscale value ≤ 150; tomatoes: 60≤grayscale value ≤ 180; Xiangli pears: 60≤grayscale value ≤ 210), effectively suppressing peel specular reflection and dark noise. Due to its rough epidermal structure, the native segmentation accuracy of Xiangli pears (Mask mAP50-95 = 0.994) is significantly superior to that of grapes (0.901) and tomatoes (0.942). This accuracy discrepancy is primarily attributed to inherent differences in the optical properties of distinct fruits rather than the enhancement effects of the model itself. Real-time performance analysis (**Figure 8**) further indicates that the average inference time of YOLOv8n-seg (**Figures 9A, D, G**) ranges from 15.38 ms (Xiangli pears) to 17.81 ms (grapes); the mask scaling processing time (**Figures 9B, E, H**) spans 5.30 ms (tomatoes) to 7.06 ms (grapes); and the thresholding processing time (**Figures 9C, F, I**) varies between 5.28 ms (Xiangli pears) and 7.14 ms (grapes). Comprehensive analysis reveals that the average total processing time per frame for grapes, tomatoes, and Xiangli pears is 32.01 ms, 26.52 ms, and 26.09 ms, respectively.

### 3.2 Performance of cross-species sugar content universal prediction model

**Figure 10:**
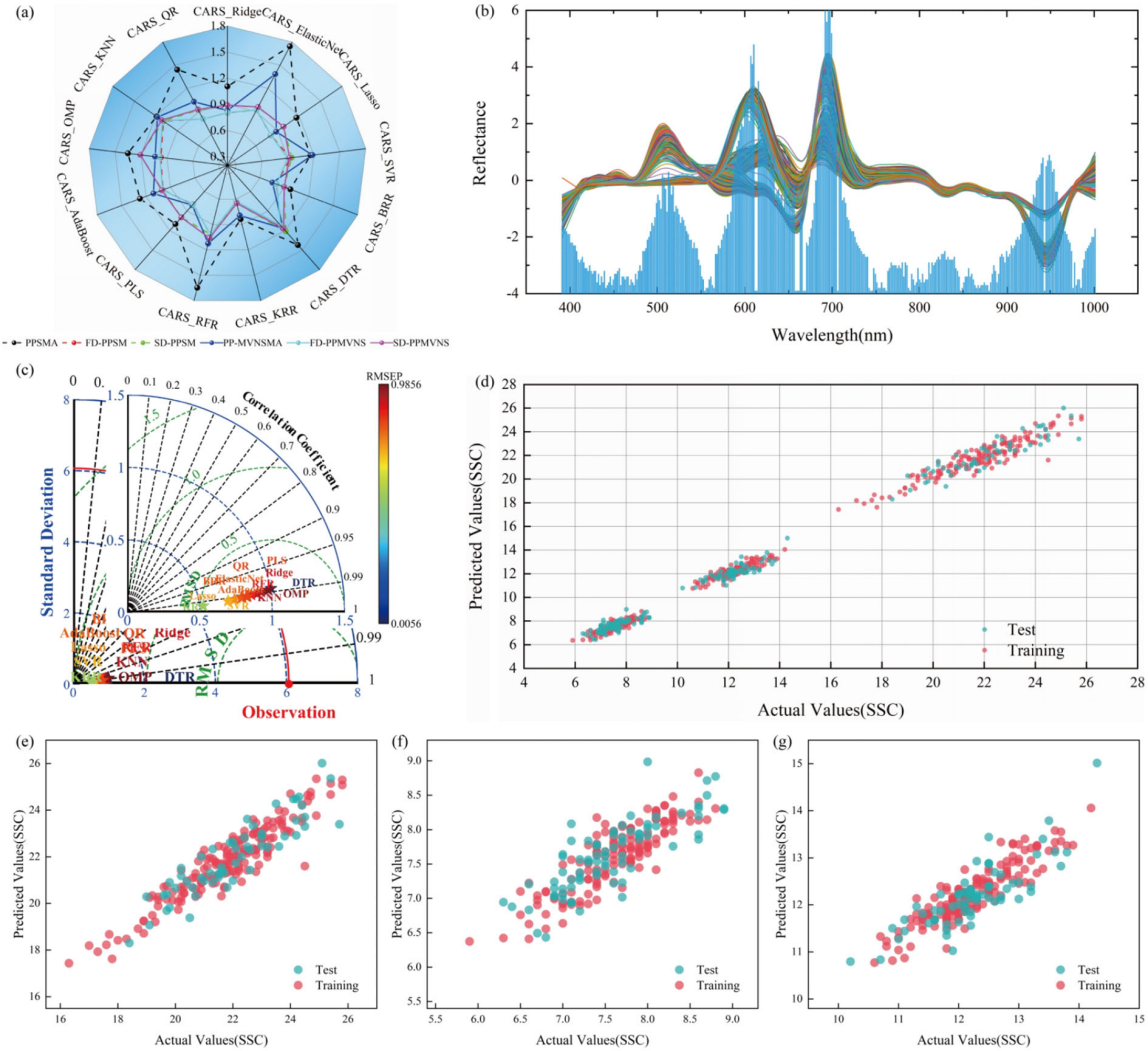
Combined prediction analysis of spectral data for multiple fruits. **(A)** Feature Extraction Performance Analysis, **(B)** Feature Band Contribution Distribution, **(C)** Regression Prediction Performance Analysis, **(D)** Multi-class Prediction Effect Analysis, **(E)** Grape Prediction Effect, **(F)** Tomato Prediction Effect, **(G)** Xiangli Pear Prediction Effect.

By integrating six types of spectral features (PPSMA, PP-MVNSMA, FD-PPSM, FD-PPMVNS, SD-PPSM, SD-PPMVNS) from grapes, tomatoes, and Xiangli pears, a cross-species universal SSC (soluble solids content) prediction model was constructed. The Competitive Adaptive Reweighted Sampling (CARS) algorithm was employed to screen the optimal feature subsets, and 13 regression models were integrated for weight updating: linear models updated weights based on the absolute values of regression coefficients; support vector machines and tree-based models adopted feature importance metrics; and K-nearest neighbors (KNN) updated weights according to feature contribution degrees. As illustrated in **Figures 10A**, under the FD-PPSM spectral data, the CARS-KRR (Kernel Ridge Regression) model achieved the optimal feature selection performance (sampling time = 1, RMSE = 0.6254°Brix, number of selected features = 346). The spatial distribution of feature contribution (**Figure 10B**) indicated that the spectral wavelength around 700 nm reached the peak contribution to SSC prediction. Based on the optimized features, 13 regression models were trained to predict soluble solids content. As analyzed in **Figures 10C, the** KRR model exhibited the best prediction performance, achieving prediction errors of 0.7565°Brix (R² = 0.8116) for grapes, 0.3672°Brix (R² = 0.6393) for tomatoes, and 0.4311°Brix (R² = 0.6625) for Xiangli pears on the test sets (**Table 2**). The overall prediction performance of the three fruit species showed a significant correlation with the true SSC values (**Figure 10D**), while **Figures 10E-G** present the localized analysis of the prediction results for grapes, tomatoes, and Xiangli pears, respectively.

**Table 2:** Combined prediction effect of spectral data for multiple fruits.

Sample	Spectral Data	Prediction method	Training set			Test set		
			R_c_^2^	RMSE_c_	MAE	R_p_^2^	RMSE_p_	MAE
Grapes	FD-PPSM	KRR	0.8735	0.6323	0.4934	0.8116	0.7565	0.5938
Tomatoes			0.7427	0.2677	0.2201	0.6393	0.3672	0.2840
Xiangli pears			0.8125	0.3049	0.2421	0.6625	0.4311	0.3478

### 3.3 Performance of single-species sugar content specialized prediction model

**Figure 11:**
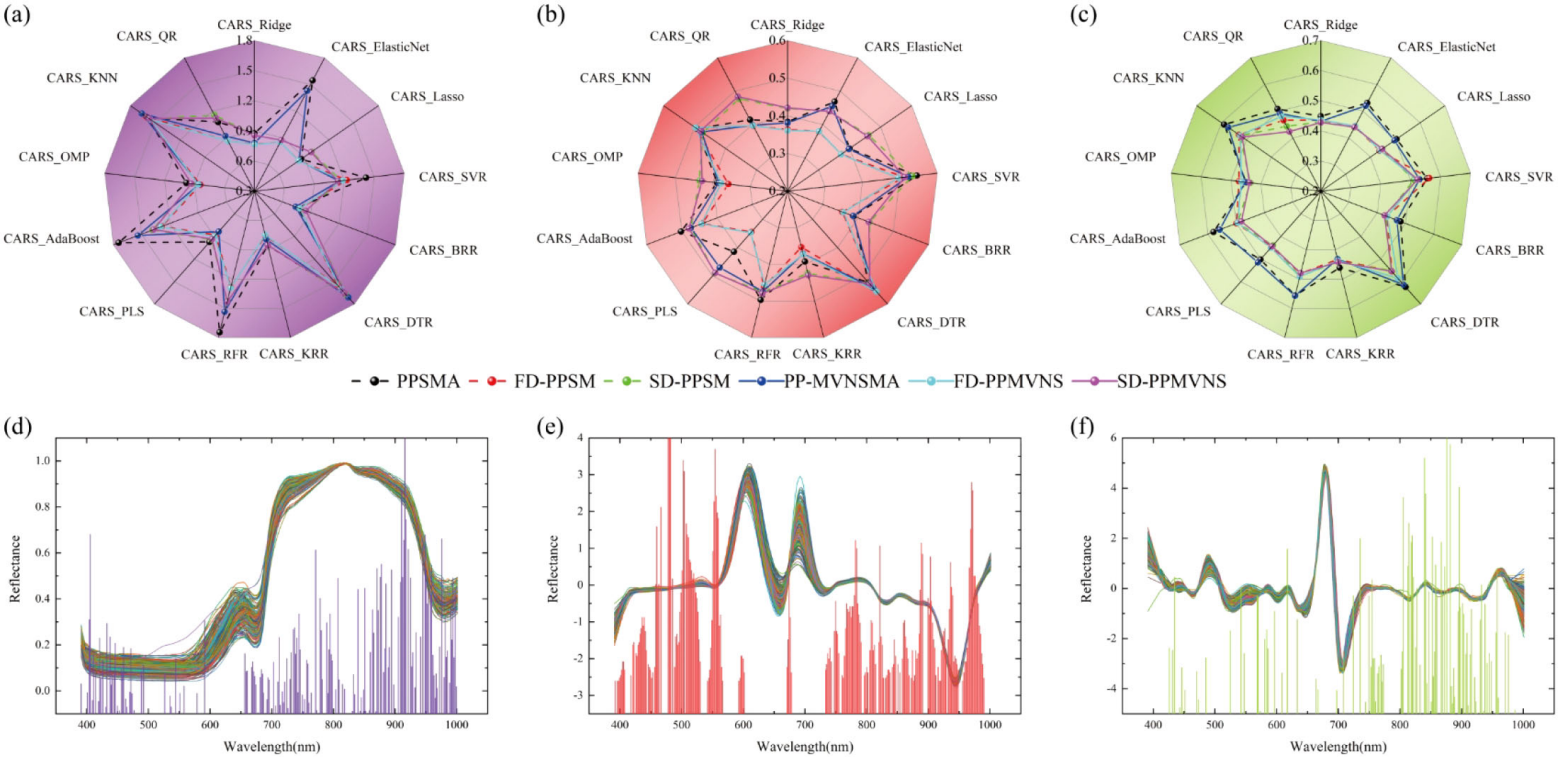
Optimal feature selection analysis for single fruits. **(A)** Feature Extraction Performance Analysis for Grapes, **(B)** Feature Extraction Performance Analysis for Tomatoes, **(C)** Feature Extraction Performance Analysis for Xiangli Pears, **(D)** Feature Band Contribution Distribution for Grapes, **(E)** Feature Band Contribution Distribution for Tomatoes, **(F)** Feature Band Contribution Distribution for Xiangli Pears.

To address the differences in optical properties among grapes, tomatoes, and Xiangli pears, specialized sugar content prediction models were constructed separately for each species. Feature optimization was performed using the Competitive Adaptive Reweighted Sampling (CARS) algorithm combined with 13 regression models, enabling independent optimization of subsets for six types of spectral features (PPSMA, PP-MVNSMA, FD-PPSM, FD-PPMVNS, SD-PPSM, SD-PPMVNS). As illustrated in **Figures 11A–C**, the CARS-Bayesian Ridge Regression (CARS-BRR) model achieved the optimal feature selection performance for grapes under the PP-MVNSMA spectral data (number of selected features = 164); for tomatoes, the CARS-Partial Least Squares (CARS-PLS) model exhibited the best feature selection performance under the FD-PPSM spectral data (number of selected features = 207); and for Xiangli pears, the CARS-Quantile Regression (CARS-QR) model delivered the optimal feature selection performance under the SD-PPMVNS spectral data (number of selected features = 96) (**Table 3**). The spatial distribution of spectral feature contribution for the optimized subsets is presented in **Figures 11D–F**.

**Table 3:** Optimal feature selection performance for single fruits.

Sample	Spectral data	Model	Optimal sampling frequency	Number of features	RMSE
Grapes	PP-MVNSMA	CARS-BRR	17	164	0.7382
Tomatoes	FD-PPSM	CARS-PLS	12	207	0.3444
Xiangli pears	SD-PPMVNS	CARS-QR	2	96	0.4235

**Figure 12:**
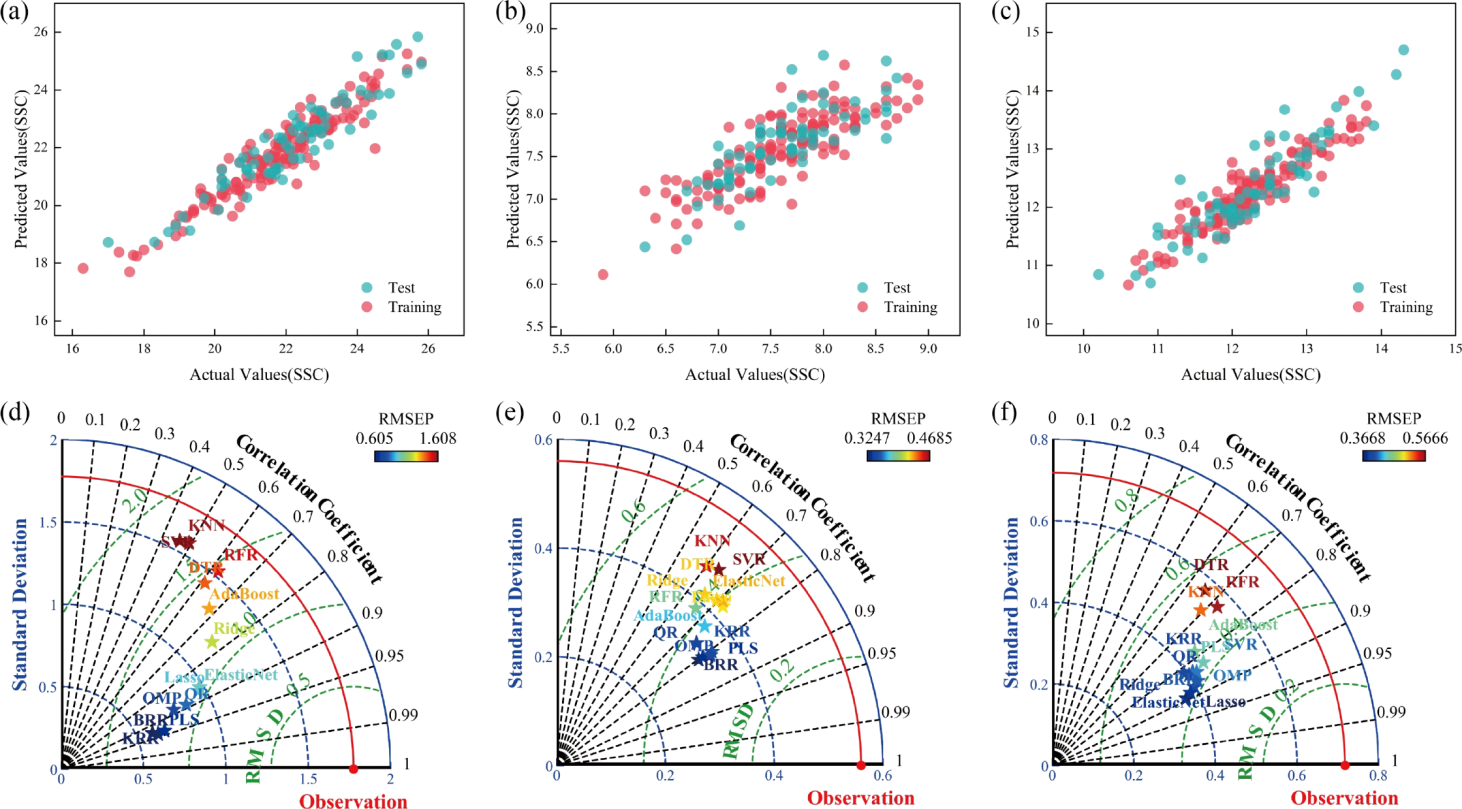
Prediction Analysis of Spectral Data for Single Fruits. **(A)** Grape Prediction Effect, **(B)** Tomato Prediction Effect, **(C)** Xiangli Pear Prediction Effect, **(D)** Regression Prediction Performance Analysis for Grapes, **(E)** Regression Prediction Performance Analysis for Tomatoes, **(F)** Regression Prediction Performance Analysis for Xiangli Pears.

Based on the optimized feature subsets, 13 regression models were trained for SSC prediction. As shown in **Figures 12D–F**, the Kernel Ridge Regression (KRR) model achieved the best prediction performance on the grape test set (RMSE = 0.6229°Brix, R² = 0.8838); the BRR model outperformed others on the tomato test set (RMSE = 0.3247°Brix, R² = 0.6430); and the Lasso Regression model delivered the optimal result on the Xiangli pear test set (RMSE = 0.3668°Brix, R² = 0.7987) (**Table 4**). A key finding indicates that Spectral Feature Spatial Heterogeneity (SSH) is a critical mechanism governing cross-species prediction. Differences in the spatial distribution of feature wavelength contribution degrees among the three fruit species reveal the intrinsic impact of optical properties on model generalization. **Figures 12A–C** display the prediction results for grapes, tomatoes, and Xiangli pears, respectively. Notably, the prediction accuracy of each specialized model for its target species is superior to that of the cross-species universal model, further verifying the adaptability of specialized models to the reflection heterogeneity of fruit surfaces.

**Table 4:** Prediction effect of spectral data for single fruits.

Sample	Spectral data	Prediction method	Training set			Test set		
			R_c_^2^	RMSE_c_	MAE	R_p_^2^	RMSE_p_	MAE
Grapes	PP-MVNSMA	KRR	0.8951	0.5610	0.4291	0.8838	0.6229	0.5218
Tomatoes	FD-PPSM	BRR	0.6590	0.3299	0.2657	0.6430	0.3247	0.2641
Xiangli pears	SD-PPMVNS	Lasso	0.8526	0.2560	0.1961	0.7987	0.3668	0.2739

### 3.4 Real-time performance verification of system deployment

In the system deployment test conducted under a simulated production line environment, the average total processing time for 226 grape samples, 221 tomato samples, and 223 Xiangli pear samples was 550.15 ms, 403.48 ms, and 769.01 ms, respectively. As illustrated in **Figure 8**, for the grape processing workflow: the first stage (encompassing data reshaping, spectral data extraction, pseudo-RGB generation, and feature extraction) consumed 260.49 ms; the second stage (target recognition, mask segmentation, and noise suppression) took 32.01 ms; the third stage (coordinate extraction, spectral data extraction, and normalization processing for a single ROI) required 194.22 ms; the fourth stage (glob-al loading of feature bands and models) lasted 59.67 ms; and feature extraction and pre-diction for a single ROI only took 3.76 ms. For tomatoes: the first stage took 244.88 ms, the second stage 26.52 ms, the third stage (single ROI processing) 116.55 ms, the fourth stage (global loading) 9.10 ms, and single ROI prediction 6.43 ms. Due to the large surface volume and significant optical heterogeneity of Xiangli pears, the third stage (single ROI processing) time increased to 470.70 ms; the time consumption of the remaining stages was as follows: 245.84 ms for the first stage, 26.09 ms for the second stage, 20.14 ms for the fourth stage (global loading), and 6.24 ms for single ROI prediction. The results indicate that the core system process meets real-time requirements (per-frame processing time< 1 second). However, the optical complexity of Xiangli pears highlights the necessity of further optimizing spectral processing algorithms to enhance cross-species adaptability.

## 4 Discussion

The proposed HyperVision-HSI framework successfully addresses the core bottlenecks of hyperspectral imaging technology in non-destructive SSC detection of cross-species fruits through a spectral-spatial adaptive decoupling mechanism. Experimental results demonstrate that the framework achieves high-precision SSC prediction across three fruit species with significantly distinct optical properties (grapes, tomatoes, and Xiangli pears), yielding root mean square errors (RMSE) of 0.62°Brix, 0.32°Brix, and 0.37°Brix, respectively, with a single-frame processing time of less than 1 second. This performance effectively meets the dual requirements of real-time responsiveness and accuracy in agricultural industrial scenarios, providing an efficient and feasible technical pathway for cross-species non-destructive detection in agricultural industrialization.

The core innovation of HyperVision-HSI lies in the integration of dynamic ROI localization, dual-channel spectral radiometric calibration, and a classification-guided light-weight model dynamic invocation architecture. Dynamic ROI localization employs centripetal scaling of mask centroids (scaling factor s = 0.18) and species-adaptive grayscale thresholds (grapes: 30–150; tomatoes: 60–180; Xiangli pears: 60–210), which effectively suppresses the interference of peel specular reflection and dark noise on spectral data, significantly enhancing the purity of biological feature regions. This design successfully overcomes the inherent limitations of traditional methods (e.g., threshold segmentation or morphological operations), which are highly sensitive to illumination variations and fruit morphology, thereby resolving the issue of dynamic localization distortion in biological feature regions. Furthermore, dual-channel radiometric calibration (based on radiometric correction and maximum normalization) significantly mitigates the spectral-spatial coupling effects induced by sensor dark current drift and light fluctuations, laying a solid da-ta foundation for the model’s generalization capability.

In terms of modeling strategy, this study pioneers a full-process technical chain of “classification-localization decoupling-feature analysis-prediction analysis” and dynamically invokes specialized regression models according to the optical properties of different fruits (grapes: KRR; tomatoes: BRR; Xiangli pears: Lasso). Experiments indicate that specialized models outperform universal models in single-species SSC prediction (e.g., grape RMSE reduced to 0.62°Brix), while universal models, despite their cross-species applicability, still exhibit limitations when handling optical heterogeneity. This phenomenon reveals that Spectral Feature Spatial Heterogeneity (SSH) is a critical factor influencing the cross-species generalization ability of models, providing important insights for the design and optimization of future models tailored to differences in optical properties.

It was observed that the processing time for Xiangli pears (769.01 ms) was higher than that for grapes (550.15 ms) and tomatoes (403.48 ms) due to their rough epidermis and significant optical heterogeneity, which highlights the challenges posed by complex optical properties to the real-time performance of the system. Although HyperVision-HSI demonstrated excellent performance under controlled experimental conditions, transitioning to real-world open agricultural industrial scenarios still faces multiple tests. First, environmental robustness is the primary direction for future optimization. This study is currently based on a stable halogen light source; however, in actual industrial sites, ambient stray light (such as sunlight fluctuations or factory lighting) may interfere with the dynamic range of the sensor and affect the benchmarks of dual-channel calibration. Therefore, developing deep learning algorithms with automatic ambient light compensation capabilities, or integrating active light-shielding devices, will be key to enhancing the system’s adaptability to non-standard environments.

Furthermore, although this study covers three types of fruits with typical optical properties, it has not yet fully addressed all biological morphologies (such as fruits with stems, leaves, or extremely irregular shapes), nor the deep impact of different origins and maturity spans on Spectral Feature Spatial Heterogeneity (SSH). Future research will focus on constructing multi-species hyperspectral databases across seasons and regions, and introducing Transfer Learning techniques to achieve rapid adaptation of the model to new species with minimal samples. Meanwhile, to meet the demand for comprehensive quality assessment in actual production, extending the framework from single soluble solids content (SSC) detection to a Multi-task Learning architecture—enabling the simultaneous prediction of sugar content, acidity, firmness, and surface defects—is also an inevitable trend to increase the throughput of detection information.

## 5 Conclusions

To address the issues of biological feature region localization distortion and spectral-spatial coupling interference in the non-destructive detection of soluble solids content (SSC) in cross-species fruits, this study proposes the HyperVision-HSI framework. The core findings are summarized as follows:(1) A three-level collaborative decoupling strategy was constructed to break through the bottlenecks in cross-species detection. By integrating dynamic ROI localization (YOLOv8n-seg + mask centroid centripetal scaling + adaptive thresholds), dual-channel spectral calibration (radiometric calibration + maximum normalization), and species-specific model matching, species-specific noise was effectively suppressed. The SSC prediction errors for grapes, tomatoes, and Xiangli pears were reduced to as low as 0.62°Brix, 0.32°Brix, and 0.37°Brix, respectively, providing a standardized technical interface for unified multi-species detection.(2) The key role of Spectral Feature Spatial Heterogeneity (SSH) was revealed. The spatial heterogeneity of spectral feature distribution driven by differences in fruit epidermal structures is the core mechanism restricting the generalization ability of cross-species models. It is confirmed that SSC prediction must adapt to species-specific optical properties, offering a new theoretical perspective for research on the correlation between bio-optical characteristics and spectral responses.(3) An industrial balance between high accuracy and real-time performance was achieved. The framework’s single-frame processing time is less than 1 second (grapes: 550.15 ms, tomatoes: 403.48 ms, Xiangli pears: 769.01 ms), breaking through the industry limitation that accuracy and real-time performance are difficult to synergize in traditional cross-species detection. This provides an extensible technical paradigm for agricultural phenomics research and industrial online sorting. Future research will expand to more horticultural fruits such as apples and citrus, and combine lightweight deep learning networks to optimize the spectral feature extraction module, further enhancing detection robustness in complex field scenarios.

## Data Availability

The original contributions presented in the study are included in the article/supplementary material. Further inquiries can be directed to the corresponding author.
